# Private Nursing Fees

**Published:** 1921-01-29

**Authors:** 


### THE MATRONS' AND SISTERS' DEPARTMENT.

### PRIVATE NURSING FEES.

THE scale of three and a-half guineas a week suggested by the College of Nursing as a proper minimum for private nurses has aroused a considerable stir. The implication is that fever, mental, and midwifery nurses are to receive as at present at least an extra guinea per week, and that other extras, such as massage or the requirement that the nurse shall wear mufti, etc., run the fees up to five guineas or more. When to this is added the cost of the nurse's board and quite possibly of extra domestic assistance, it becomes evident that none but the opulent will be able to afford the luxury of a trained nurse. Taking the suggestion in its most favourable light it will, if generally accepted, tend to reduce considerably the area of employment for fully certified nurses, and make a corresponding increase in the demand for the partially trained.

The new scale of salaries brought out by the College does not at first sight allow for the present change in the value of money which has so powerfully affected the pay of nurses. The private nurse, who receives board, lodging, uniform, and laundry as part of her emoluments, is unaffected by high prices as compared with the non-resident nurse, who has to meet every expense (except perhaps that of uniform) out of her salary. The ordinary private nurse without extra qualifications who is to receive a minimum of £176 a year, for forty-eight weeks work, is boarded and lodged, etc., during the whole time excepting her four weeks' holiday; does she not appear to be in a far better position than that of her confrère in a non-residential post, who boards and lodges herself all the year round and has only £74 more? This computation, however, does not allow for the chances of private nursing. The nurse is probably employed only about forty-two weeks in the year; she would earn on the three and a-half guinea scale £163, which approximates to the earnings of the non-resident nurse, for every one seems now to agree that at least £100 a year must be reckoned for the cost of living. But it will be asked, Will the public be prepared to pay their nurses on a basis which allows of their taking ten weeks' holiday in the year? Surrendering all their Sundays as they do during their time on duty private nurses are entitled, however, to have these made up for them, and forty-one Sundays amount to close on six weeks. Add to these weeks the ordinary four weeks' holiday universally allowed and we find that in order to bring the private nurse into a position of equality with the non-resident district nurse or public health nurse three and a-half guineas a week is not too much.

It is somewhat on these lines that we imagine the College computation to have been made, and if we take it that £150 a year, plus board and lodging, is a fair salary for the private as for the public health nurse, the three and a-half guineas a week minimum is indicated.

It by no means follows that it can be obtained. At the present moment we know of no important co-operation of nurses or hospital institutes which has adopted it for their members. It looks rather as if private nursing were passing through a difficult stage to match the perplexities of the patients. It must never be forgotten that the habitual employers of nurses are in large measure people of fixed income feeling the pinch of higher prices, higher rents, exorbitant taxation. The nurse's food is costing the patient about three times what it used to do. Service is difficult to find at any price, laundry rates are well-nigh preposterous. The very room which the nurse occupies has doubled in cost to the householder. The nurse's fee has already increased from two to three guineas. Will she be well advised to press for the extra half guinea a week? This is what private nurses themselves are anxiously debating.

The unmistakable rise in the economic value of nursing is often confounded with the rise in the cost of living. In some quarters the issue has been confused by conferring all increases as "war bonus." But it is generally admitted that salaries will never sink to their former level even should prices descend more rapidly than seems now probable. Salaries and prices hang together and must do so because prices regulate the value of the salary. Judged by the present value of the sovereign, which has sunk in purchasing power to something like 7s. 6d., the increases in salaries which have marked the last few years are obviously inadequate and the College is doing good service in bringing this to notice. The private nurse seven years ago earning two guineas a week was in a better position than she holds now when she earns three. All the same it may be better for the future of private nursing that fees should not rise in this transition time in full proportion to the high cost of living. Should the public get out of the habit of employing nurses in their own homes and resort instead to nursing homes or pay beds in hospital, it would mean a change from which the nursing profession would suffer acutely.

We should welcome the views of private nurses on this difficult problem.

